# Donepezil for dementia with Lewy bodies: meta‐analysis of multicentre, randomised, double‐blind, placebo‐controlled phase II, III, and, IV studies

**DOI:** 10.1111/psyg.13101

**Published:** 2024-03-04

**Authors:** Etsuro Mori, Manabu Ikeda, Megumi Ohdake

**Affiliations:** ^1^ Department of Behavioural Neurology and Neuropsychiatry Osaka University United Graduate School of Child Development Osaka Japan; ^2^ Department of Psychiatry Osaka University Graduate School of Medicine Osaka Japan; ^3^ Clinical Planning and Development Department Medical HQs, Eisai Co. Ltd Tokyo Japan

**Keywords:** cholinesterase inhibitors, donepezil, Lewy body dementia, meta‐analysis

## Abstract

**Background:**

Current evidence for the management of symptoms associated with dementia with Lewy bodies (DLB) using donepezil is limited. We conducted a meta‐analysis of three randomised controlled trials of donepezil in patients with DLB to investigate the overall efficacy of donepezil on Mini‐Mental State Examination (MMSE), Neuropsychiatric Inventory (NPI), and Clinician's Interview‐Based Impression of Change‐plus Caregiver Input (CIBIC‐plus).

**Methods:**

A meta‐analysis was performed using the data of 312 patients administered placebo or 10 mg donepezil. Overall mean score differences for MMSE, NPI‐2, and NPI‐10 from baseline to week 12 and their 95% confidence intervals (CI) were estimated. For CIBIC‐plus, which was transformed from a seven‐point grade to a dichotomous outcome (improvements/no improvements), odds ratio (OR) and its 95% CI were estimated. Random‐effects models were used, and heterogeneity was evaluated using the Cochrane's Q test and I^2^ statistic.

**Results:**

Heterogeneity was suspected for NPI‐2 (*P* < 0.05; I^2^ = 87.2%) and NPI‐10 (*P* < 0.05; I^2^ = 67.7%) while it was not suspected for MMSE (*P* = 0.23; I^2^ = 32.4%) and CIBIC‐plus (*P* = 0.26; I^2^ = 19.8%). The overall mean MMSE score difference (mean difference: 1.50; 95% CI, 0.67–2.34) and the overall odds of improving CIBIC‐plus (OR: 2.20; 95% CI, 1.13–4.26) from baseline to week 12 were higher in the donepezil group than in the placebo group.

**Conclusion:**

Results of our meta‐analysis indicated overall efficacy of donepezil on cognitive impairment and global clinical status in patients with DLB.

## INTRODUCTION

Donepezil (Aricept®, Eisai Co. Ltd., Tokyo, Japan), a type of cholinesterase inhibitors (ChEIs), is the world's first drug, approved for dementia with Lewy bodies (DLB) in September 2014 in Japan. Prior to the approval, multicentre, randomised, double‐blind, and placebo‐controlled phase II and III clinical trials demonstrated the efficacy of donepezil in patients with DLB on cognitive impairment, which was assessed using the Mini‐Mental State Examination (MMSE).^1^, ^2^, ^3^ The results of these studies led to donepezil's approved indication for DLB, contingent upon the evaluation of its efficacy on global clinical status in a post‐marketing placebo‐controlled clinical study.

After the approval, a 12‐week multicentre, randomised, double‐blind, and placebo‐controlled phase IV study was conducted to evaluate the efficacy and safety of donepezil, focusing on the global clinical status.^4^ Although the study did not find the superiority of donepezil on global clinical status over placebo using Clinician's Interview‐Based Impression of Change‐plus Caregiver Input (CIBIC‐plus), significant improvement was found in cognitive function, which was assessed as one of the four CIBIC‐plus domains.^4^ The study also evaluated the efficacy on cognitive impairment using the MMSE and behavioural and neuropsychiatric symptoms using the Neuropsychiatric Inventory (NPI)‐2 and NPI‐10 as secondary endpoints.^4^, ^5^, ^6^ The level of cognitive impairment improved in the donepezil group, whereas behavioural and neuropsychiatric symptoms did not improve.

Systematic reviews indicate that current evidence for the symptomatic management of DLB with acetylcholinesterase inhibitors is strong, although still limited to a few relatively small randomised controlled trials (RCTs).^7^, ^8^ The efficacy of donepezil on MMSE was consistently shown in our previous RCTs of donepezil, whereas its efficacy on NPI and CIBIC‐plus was not consistently shown.^2^, ^3^, ^4^ Hence, it would be meaningful to compile our RCT data and comprehensively assess and understand the general trend of efficacy of donepezil. The objective of this study was to investigate the overall efficacy of donepezil on the MMSE, NPI, and CIBIC‐plus in patients with DLB, based on a meta‐analysis of three RCTs of donepezil previously conducted in Japan.^2^, ^3^, ^4^

## METHODS

### Data source

The data used in this study were collected from three RCTs of donepezil for probable DLB carried out in Japan, with similar design to the multicentre, randomised, double‐blind, and placebo‐controlled trial (Table 1).^2^, ^3^, ^4^ Patients were diagnosed with probable DLB according to the 1996 criteria in phase II and III studies^9^ and according to the 2005 revised criteria in a phase IV study.^10^

**Table 1:** Details of RCT studies of donepezil previously conducted among patients with DLB in Japan and assessment variables that were relevant in the meta‐analysis

Study phase	Study design	The start of recruitment period	Aim	Participating institutions	Participants	Endpoints
Phase II^2^	12‐week multicentre, randomised, double‐blind, and placebo‐controlled trial	October 2007	To investigate the efficacy and safety of donepezil at 3, 5, and 10 mg/day over placebo	48	135	MMSE NPI (NPI‐2, NPI‐10) CIBIC‐plus Others^†^
Phase III^3^	16‐week multicentre, randomised, double‐blind, and placebo‐controlled trial	February 2011	To confirm the superiority and evaluate safety of donepezil at 5 and 10 mg/day over placebo	72	138	MMSE NPI (NPI‐2, NPI‐10) Others^‡^
Phase IV^4^	12‐week multicentre, randomised, double‐blind, and placebo‐controlled trial	March 2015	To assess efficacy and safety of donepezil at 10 mg/day over placebo	54	151	MMSE NPI (NPI‐2, NPI‐10) CIBIC‐plus Others^§^

RCT, randomised controlled trial; DLB, dementia with Lewy bodies; MMSE, Mini‐Mental State Examination; NPI, Neuropsychiatric Inventory; CIBIC‐plus, Clinician's Interview‐Based Impression of Change‐plus Caregiver Input.

^†^ Other assessment variables investigated in this study included Wechsler Memory Scale‐Revised (WMS‐R) attention/concentration, category fluency, letter fluency, Wechsler Adult Intelligence Scale (WAIS‐III) symbol digit modalities, Visual Perception Test for Agnosia (VPTA) form recognition, NPI‐4, Zarit Caregiver Burden Interview (ZBI), Unified Parkinson's Disease Rating Scale (UPDRS) part III, and adverse events.

^‡^ Other assessment variables investigated in this study included ZBI, UPDRS part III, and adverse events.

^§^ Other assessment variables investigated in this study included adverse events.

Inclusion criteria of three studies^2^, ^3^, ^4^ were outpatients aged ≥50 years with mild to moderate–severe dementia (a score of 10–26 on the MMSE and Clinical Dementia Rating (CDR) ≥ 0.5) and behavioural and psychiatric symptoms (for phase II and III: NPI‐plus ≥8 (NPI‐plus comprises 12 items of the original 10 NPI items, sleep,^5^, ^6^ and cognitive fluctuation reported as Cognitive Fluctuation Inventory^11^, ^12^); for phase III, NPI‐2 ≥ 1 (NPI‐2 comprises hallucinations and cognitive fluctuation^2^); for phase IV, NPI‐2 ≥ 2). Eligible patients were required to have caregivers who routinely stayed with the patients at least 4 h a day and 3 days a week and agreed to provide information, assist with treatment compliance, and escort patients to required hospital visits during each study.

Exclusion criteria of three studies^2^, ^3^, ^4^ were Parkinson's disease diagnosed at least 1 year prior to the onset of dementia; focal vascular lesions on magnetic resonance imaging or computed tomography scan that might cause cognitive impairment; other neurological or psychiatric diseases; complications or a history of severe gastrointestinal ulcer, severe asthma, or obstructive pulmonary disease; systolic hypotension (<90 mmHg); bradycardia (<50 beats/min); sick sinus syndrome; atrial or atrioventricular conduction block; QT interval prolongation (≥450 msec); severe parkinsonism (Hoehn and Yahr stage ≥ IV),^13^ and treatment with ChEIs, or any investigational drug within 3 months prior to screening. ChEIs, antipsychotics, and central anticholinergics (for phases II and III, including anti‐Parkinson drugs (other than levodopa or dopamine agonists)) were not allowed during studies.

### Ethical considerations

All three studies were conducted in accordance with the Declaration of Helsinki and Good Clinical Practice (GCP). In accordance with GCP, each study protocol was reviewed and approved at each participating centre by the institutional review board. In addition to GCP, a phase IV study accorded the Good Post‐marketing Study Practice.The phase IV study was conducted using a placebo‐controlled design when donepezil was already on the market. Thus, considering ethical aspects of this study, disease education and caregiving guidance were provided for all participants and caregivers at each visit to a designated hospital after informed consent was obtained.

### Assessment efficacy variables

Cognitive impairment was assessed using the MMSE prior to donepezil administration (baseline) and 12 weeks after the administration (week 12). The MMSE comprises 11 subitems, and each subitem evaluates different cognitive domains, such as orientation, memory, attention, and construction.^1^ The total score ranges 0–30.

Behavioural and neuropsychiatric symptoms were assessed using the NPI‐2 and NPI‐10 at baseline and week 12. The NPI‐2 comprises subitems of hallucinations and cognitive fluctuation.^2^ The NPI‐10 comprises subitems of delusions, hallucinations, agitation/aggression, dysphoria, anxiety, euphoria, apathy, disinhibition, irritability/lability, and aberrant motor behaviour.^5^, ^6^ Each NPI subitem is scored based on frequency and severity, and each score ranges 0–12. The total NPI‐2 and NPI‐10 scores are calculated by adding two or 10 scores of each subitem.

Changes in global clinical status from baseline were assessed at week 12 using the CIBIC‐plus. The CIBIC‐plus comprehensively evaluates changes in global clinical status from the baseline in terms of four domains: general condition, cognitive function, behaviours, and activities of daily living, during semi‐structured interviews.^14^ The CIBIC‐plus is rated at a seven‐point grade (1, marked worsening; 2, moderate worsening; 3, minimal worsening; 4, no change; 5, minimal improvement; 6, moderate improvement; and 7, marked improvement). The global clinical status at baseline was assessed using the Clinician's Interview‐Based Impression of Severity (CIBIS). The CIBIS evaluates the same domains as the CIBIC‐plus and is rated at a seven‐point grade (1, normal; 2, borderline mentally ill; 3, mildly mentally ill; 4, moderately mentally ill; 5, markedly mentally ill; 6, severely mentally ill; and 7, most extremely ill).

### Statistical analysis

Patient and clinical characteristics were descriptively analyzed with mean ± SD or number and percentage, as appropriate.

A meta‐analysis was performed based on data of patients administered placebo or 10 mg donepezil. Data at week 12 were used, and the last observation carried forward (LOCF) approach was used for missing data at week 12. The overall mean score differences for the MMSE, NPI‐2, and NPI‐10 from baseline to week 12 and their 95% confidence intervals (CI) were estimated based on data from all three studies. For the CIBIC‐plus, the odds ratio (OR) and 95% CI were estimated to simplify the interpretations based on data from phase II and IV studies (the CIBIC‐plus was not set as an endpoint in the phase III study). For the analysis, the original seven‐point grade score of CIBIC‐plus was transformed to a dichotomous outcome with improvements (marked, moderate, and minimal improvement) and no improvements (no changes and minimal to marked worsening).

The meta‐analysis was performed using the random‐effects models, and heterogeneity across studies was evaluated using the Cochrane's Q test and I^2^ statistic. All tests were two‐sided with a significance level of 0.05. The software package SAS Release 9.3 (SAS Institute, Cary, NC, USA) was used for all analyses.

## RESULTS

### Patient and clinical characteristics

Table 2 summarises patient and clinical characteristics. The age of participants were similar among studies (phase II, placebo: 78.6 ± 4.7 years old, 10 mg donepezil: 78.6 ± 6.1 years old; phase III, 77.2 ± 6.1 years old, 77.7 ± 6.8 years old; phase IV, 77.6 ± 7.15 years old, 78.0 ± 6.44 years old). Males were less represented in the phase II study (28.1%, 11.1%) than in the other two studies (phase III, 38.6%, 42.9%; phase IV, 39.5%, 45.3%); body weights of patients were lighter in the phase II study than those in the other two studies (phase II, 47.53 ± 9.02 kg, 44.94 ± 9.15 kg; phase III, 50.15 ± 10.75 kg, 51.72 ± 9.89 kg; phase IV, 55.17 ± 11.473 kg, 52.41 ± 11.396 kg). Of the core features of DLB diagnosis, visual hallucinations were more prevalent in the phase II and III studies (phase II, 87.5%, 80.6%; phase III, 95.5%, 79.6%) than in the phase IV study (67.1%, 65.3%), whereas the prevalence of cognitive fluctuations and parkinsonism were comparable across the three studies.

**Table 2:** Patient details and clinical characteristics of those administered placebo or 10 mg donepezil at baseline

	Study phase					
	Phase II^2^		Phase III^3^		Phase IV^4^	
	Placebo	10 mg	Placebo	10 mg	Placebo	10 mg
	(*n* = 32)	(*n* = 36)	(*n* = 44)	(*n* = 49)	(*n* = 76)	(*n* = 75)
Age, years	78.6 ± 4.7	78.6 ± 6.1	77.2 ± 6.1	77.7 ± 6.8	77.6 ± 7.15	78.0 ± 6.44
Sex, male, *n* (%)	9 (28.1)	4 (11.1)	17 (38.6)	21 (42.9)	30 (39.5)	34 (45.3)
Weight, kg	47.53 ± 9.02	44.94 ± 9.15	50.15 ± 10.75	51.72 ± 9.89	55.17 ± 11.473	52.41 ± 11.396
Duration of dementia, years	2.85 ± 2.26^†^	1.63 ± 1.44	2.0 ± 2.3	2.3 ± 1.9	2.34 ± 1.877	2.15 ± 1.960
Cognitive fluctuation, *n* (%)	31 (96.9)	35 (97.2)	40 (90.9)	46 (93.9)	71 (93.4)	70 (93.3)
Visual hallucinations, *n* (%)	28 (87.5)	29 (80.6)	42 (95.5)	39 (79.6)	51 (67.1)	49 (65.3)
Parkinsonism, *n* (%)	28 (87.5)	29 (80.6)	38 (86.4)	44 (89.8)	64 (84.2)	65 (86.7)
MMSE score	18.3 ± 4.7	19.8 ± 4.4	20.3 ± 4.2	20.3 ± 4.8	20.0 ± 4.04	20.5 ± 4.36
NPI‐2 score	6.3 ± 4.0	7.9 ± 5.4	6.9 ± 4.5	7.3 ± 4.7	5.9 ± 4.27	5.7 ± 3.98
NPI Hallucinations	3.7 ± 3.5	4.1 ± 4.0	3.3 ± 3.0	3.4 ± 3.1	2.4 ± 3.17	2.2 ± 3.08
NPI Cognitive fluctuation	2.7 ± 2.1	3.8 ± 3.4	3.7 ± 2.8	3.9 ± 2.9	3.5 ± 2.54	3.5 ± 2.50
NPI‐10 score	18.3 ± 8.9	19.5 ± 12.8	20.5 ± 15.0	16.6 ± 11.7	12.9 ± 10.41	14.4 ± 10.90
CIBIS, *n* (%)						
Normal	0 (0.0)	0 (0.0)	‐	‐	0 (0.0)	0 (0.0)
Borderline mentally ill	2 (6.3)	2 (5.6)	‐	‐	2 (2.6)	8 (10.7)
Mildly mentally ill	8 (25.0)	14 (38.9)	‐	‐	42 (55.3)	38 (50.7)
Moderately mentally ill	18 (56.3)	13 (36.1)	‐	‐	27 (35.5)	18 (24.0)
Markedly mentally ill	2 (6.3)	5 (13.9)	‐	‐	3 (3.9)	7 (9.3)
Severely mentally ill	2 (6.3)	2 (5.6)	‐	‐	2 (2.6)	4 (5.3)
Among most extremely ill	0 (0.0)	0 (0.0)	‐	‐	0 (0.0)	0 (0.0)
Caregivers' relationship to participants, *n* (%)			‐	‐		
Husband/wife	14 (43.8)	12 (33.3)	24 (54.5)	23 (46.9)	39 (51.3)	40 (53.3)
Child	17 (53.1)	22 (61.1)	19 (43.2)	24 (49.0)	32 (42.1)	28 (37.3)
Relatives other than the above	1 (3.1)	2 (5.6)	1 (2.3)	0 (0.0)	2 (2.6)	5 (6.7)
Other	0 (0.0)	0 (0.0)	0 (0.0)	2 (4.1)	3 (3.9)	2 (2.7)
Living with participants, *n* (%)	28 (87.5)	32 (88.9)	37 (84.1)	41 (83.7)	68 (89.5)	61 (81.3)
Days spent with participants per week	7.0 ± 0.2^‡^	6.9 ± 0.4^§^	6.5 ± 1.1	6.6 ± 1.1	6.6 ± 1.05	6.3 ± 1.39
Hours spent with participants per day	10.9 ± 4.0^‡^	11.8 ± 4.4^§^	10.9 ± 5.1	10.8 ± 4.6	10.9 ± 4.93	10.4 ± 4.87

Data are expressed as mean ± SD, unless otherwise specified.

MMSE, Mini‐Mental State Examination; NPI, Neuropsychiatric Inventory (NPI‐2 = hallucinations + cognitive fluctuation; NPI‐10 = delusions + hallucinations + agitation/aggression + dysphoria + anxiety + euphoria + apathy + disinhibition + irritability/lability + aberrant motor behaviour); CIBIS, Clinician's Interview‐Based Impression of Severity.

^†^ *n* = 31.

^‡^ *n* = 28.

^§^ *n* = 32.

### Meta‐analysis

**Figure 1:**
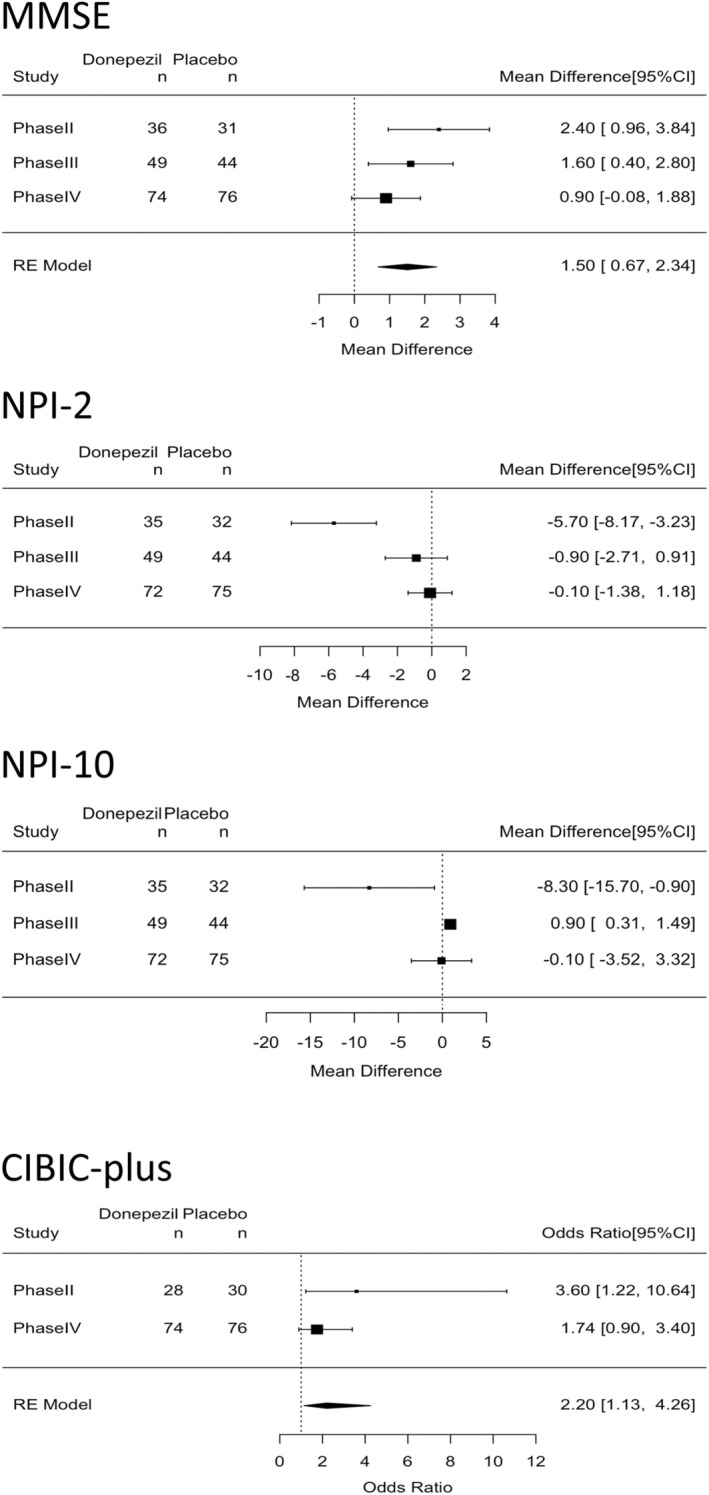
Mean score difference between donepezil and placebo groups in MMSE, NPI‐2, and NPI‐10, and odds ratio of CIBIC‐plus and associated 95% CI. MMSE, Mini‐Mental State Examination; NPI, Neuropsychiatric Inventory (NPI‐2 = hallucinations + cognitive fluctuation; NPI‐10 = delusions + hallucinations + agitation/aggression + dysphoria + anxiety + euphoria + apathy + disinhibition + irritability/lability + aberrant motor behaviour); CIBIC‐plus, Clinician's Interview‐Based Impression of Change‐plus Caregiver Input; CI, confidence interval.

Prior to the calculation of the mean score difference between the donepezil and placebo groups in the three studies, heterogeneity among the studies was tested, and heterogeneity was suspected for the NPI‐2 (Q = 15.7, df = 2, *P* < 0.05; I^2^ = 87.2%) and NPI‐10 (Q = 6.18, df = 2, *P* < 0.05; I^2^ = 67.7%) while it was not suspected for the MMSE (Q = 2.96, df = 2, *P* = 0.23; I^2^ = 32.4%) and CIBIC‐plus (Q = 1.25, df = 1, *P* = 0.26; I^2^ = 19.8%). The mean MMSE score difference from baseline to week 12 was higher in the donepezil group than in the placebo group in all three studies, and the overall mean MMSE score difference was also higher in the donepezil group (mean difference between donepezil and placebo groups: 1.50; 95% CI, 0.67–2.34, Fig. 1). The odds of improvement in the CIBIC‐plus at week 12 were higher in the donepezil group than in the placebo group in the phase II study, but such higher odds were not found in the phase IV study. The overall odds of improving the CIBIC‐plus were higher in the donepezil group than in the placebo group (OR: 2.20; 95% CI, 1.13–4.26, Fig. 1).

## DISCUSSION

This study investigated the overall efficacy of 10 mg donepezil on MMSE, NPI, and CIBIC‐plus in patients with DLB, using meta‐analysis based on data collected in three RCTs of donepezil in Japan. Based on 312 patient data, our meta‐analysis indicated efficacy of donepezil over placebo on MMSE and dichotomous CIBIC‐plus. Heterogeneity in NPI‐2 and NPI‐10 scores among studies prevented the overall efficacy assessment.

The results of the phase II and III studies, that MMSE improved both in the phase II and III studies while the CIBIC‐plus improved in the phase II study and was not assessed in the phase III study, led to donepezil's approved indication for DLB,^2^, ^3^ but conditional on the evaluation of donepezil's efficacy on global clinical status in a phase IV placebo‐controlled clinical study. The collateral condition was given because guidelines for the clinical evaluation of antidementia drugs in Europe, USA, and Japan mutually stipulate that, to gain an antidementia indication, a drug must (1) exert its effect on the core clinical feature and (2) have a clinically meaningful effect.^15^, ^16^, ^17^, ^18^ Donepezil's efficacy on MMSE was consistently indicated in all three RCT studies. In contrast, the efficacy of donepezil on the NPI‐2, NPI‐10, and CIBIC‐plus was not consistently found.^2^, ^3^, ^4^ By conducting these three RCTs, data on donepezil in DLB patients have been accumulated, enabling us to assess overall efficacy of donepezil comprehensively on the MMSE, NPI, and CIBIC‐plus in patients with DLB. Thus, our study adds meaningful results to the current evidence for management of symptoms associated with DLB.

As previously shown in each of the RCT studies of donepezil,^2^, ^3^, ^4^ we confirmed the overall efficacy of 10 mg donepezil on MMSE in this meta‐analysis. Our meta‐analysis also indicated overall efficacy of 10 mg donepezil on CIBIC‐plus, using the dichotomous CIBIC‐plus, which was transformed from the original seven‐point grade score. Although the results of this study need to be carefully interpreted, these results suggest that treatments using donepezil improved both cognitive impairment and global clinical status in patients with DLB. Symptoms of DLB include, but are not limited to, fluctuating cognition, visual hallucinations, and motor symptoms of parkinsonism, as well as cognitive impairment characterised by deficits in attention, executive function, and visual perception.^9^ Improvements in both cognitive impairment and global clinical status can be significant for patients with DLB as current evidence for managing a range of DLB symptoms is still limited^7^, ^8^ and can also be socially significant for caregivers of the patients, as caregiver burden is more substantial in patients with DLB than in those with Alzheimer's disease.^2^, ^19^

We found heterogeneity in the NPI‐2 and NPI‐10 scores among the three RCT studies. Heterogeneity among the three studies was also found in each NPI‐2 subitem score (hallucination, Q = 10.0, df = 2, *P* < 0.05; I^2^ = 79.9%; cognitive fluctuation, Q = 10.0, df = 2, *P* < 0.05; I^2^ = 80.0%). Such heterogeneity in NPI scores may have been attributable to the prolonged period from the time of phase II study to phase IV study (i.e., approximately 10 years apart as phase II and IV studies were initiated in 2007 and 2015, respectively) and the unique assessment method of NPIs. Over the past decade, the awareness for DLB had increased, and caregiving methodology had improved. Moreover, the NPI is an assessment scale implemented through interviews with caregivers only. These features may have influenced the differences in NPI‐related data across the three studies, especially because disease education and caregiving guidance were provided generously to all participants and caregivers during the phase IV study at each visit to a designated hospital. Nonetheless, reasons for heterogeneity among the three studies are uncertain. Overall efficacy of 10 mg donepezil on NPI could not be evaluated, and further studies are needed to evaluate its efficacy on behavioural and neuropsychiatric symptoms of DLB. This meta‐analysis compiled existing data collected during different phases of drug development conducted over a period of more than a decade. While we are aware that such temporal transitions may alter any potential background characteristics of the subjects, it is worthwhile to be able to show consistent trends with respect to the efficacy of donepezil from RCTs, each with a small sample size.

### Conclusion

The present study, based on a meta‐analysis of RCTs of donepezil conducted in Japan, indicated overall improvements in MMSE and dichotomous CIBIC‐plus in patients with DLB who were administered 10 mg donepezil. Although careful interpretation is required, these results suggested overall efficacy of donepezil on cognitive impairment and global clinical status in patients with DLB.

## DISCLOSURE

Author EM received unlimited research grants and personal fees from Eisai, Sumitomo Pharma, and Nihon Mediphysics during the conduct of the study. Department of Behavioural Neurology and Neuropsychiatry, Osaka University United Graduate School of Child Development to which EM is assigned is an endowment department, supported with unrestricted grants from Sosei‐Heptares and Mentis Cura. Author MI received the following: consulting fees or paid advisory boards, lectures fees, and grant support from Eisai, Sumitomo Pharma, Otsuka Pharmaceutical Co., and Eli Lilly; consulting fees or paid advisory boards from Shionogi Pharma and Fronteo; and lecture fees from Yoshitomiyakuhin, Tsumura, and Nihon Medi‐physics Co. Author MO is an employee of Eisai Co. Ltd.

## Data Availability

The data are not publicly available due to privacy or ethical restrictions.
